# Complete Genome Sequencing of *Stenotrophomonas acidaminiphila* ZAC14D2_NAIMI4_2, a Multidrug-Resistant Strain Isolated from Sediments of a Polluted River in Mexico, Uncovers New Antibiotic Resistance Genes and a Novel Class-II Lasso Peptide Biosynthesis Gene Cluster

**DOI:** 10.1128/genomeA.01433-15

**Published:** 2015-12-10

**Authors:** Pablo Vinuesa, Luz Edith Ochoa-Sánchez

**Affiliations:** Centro de Ciencias Genómicas, Programa de Ingeniería Genómica, Universidad Nacional Autónoma de México, Cuernavaca, Morelos, Mexico

## Abstract

Here, we report the first complete genome sequence of a *Stenotrophomonas acidaminiphila* strain, generated with PacBio RS II single-molecule real-time technology, consisting of a single circular chromosome of 4.13 Mb. We annotated mobile genetic elements and natural product biosynthesis clusters, including a novel class-II lasso peptide with a 7-residue macrolactam ring.

## GENOME ANNOUNCEMENT

The genus *Stenotrophomonas* currently comprises 13 validly described species (http://www.bacterio.net/stenotrophomonas.html), but genome sequences are available only for *S. maltophilia* and *S. rhizophila*. Here, we report the first and complete genome sequence of an *S. acidaminiphila* isolate. Strain ZAC14D2_NAIMI4_2 (BioProject PRJNA296415; BioSample SAMN04099006) was recovered from superficial sediments of a polluted river in Morelos, Mexico (1). Strain ZAC14D2_NAIMI4_2 was confidently classified based on a multilocus sequence analysis (1), using the 7 loci of the *S. maltophilia* scheme (2).

Genomic DNA was purified with the DNeasy blood and tissue kit (Qiagen) and sheared into ~10- to 20-kb fragments for PacBio library preparation and P6-C4 sequencing on one single-molecule real-time (SMRT) cell at the Yale Center for Genome Analysis (USA). The continuous long reads were assembled using the HGAP/Quiver-protocol in SMRT portal version 2.3.0.140936.p4 (3), resulting in an assembly with 1 contig. It was circularized by trimming the terminal repeats with Minimus2 (4), and subjected to three consecutive rounds of read remapping with the RS_Resequencing.1 module, resulting in a final assembly with a mean coverage of ~150× and 100% concordance with the reference. The size of the assembled genome is 4,138,297 bp, with a G+C content of 68.48%.

Gene calling and annotation was performed with a modified version of Prokka (5) that filters BLASTp (6) results taking query coverage (≥80%) into account, interrogates NCBI’s RefSeq prokaryotic nonredundant proteins database (http://www.ncbi.nlm.nih.gov/refseq/about/nonredundantproteins), and properly classifies ncRNAs, adding the ncRNA_class attribute. A total of 3,793 genes, 3,617 coding sequences, and 16 pseudogenes were identified. Additionally, genes for 68 tRNAs, 9 rRNAs, and 1 tmRNA were annotated, plus 20 ncRNAs, 7 riboswitches (Cobalamin, Glycine, SAM, and TPP), and 532 signal peptides. The annotation was enriched with prophage predictions suggested by the PHAST server (7), genomic islands detected by IslandViewer3 (8), and secondary metabolite biosynthesis gene cluster predictions made by antiSMASH version 3.0.4 (9). The annotation was manually curated. The presence of antibiotic resistance genes and antibiotic efflux pumps was further investigated using BLASTp (6) searches against locally maintained versions of the CARD (10) and ResFinder (11) databases, and hmmscan (12) searches against ResFam (13). Together, these analyses revealed the presence of at least 6 RND and 3 ABC antibiotic efflux pump systems, a novel putative chloramphenicol acetyltransferase (AOT14_04360) linked to the genomic island GI_Stac2, two Ambler class-A beta-lactamases, a class-B metallo-beta-lactamase, and OqxA and QnrB20 homologues, potentially conferring resistance against fluoroquinolones. No class-1 or class-2 integrons, or IS*CR* elements were found.

Four gene clusters were detected, potentially encoding for the production of a homoserine lactone, a class-III bacteriocin, an aryl polyene related to Xanthomonadin (14), and a novel class-II lasso peptide. Analysis of the precursor peptide of the latter (MNSNDNTGVHADE**VI**V**LG**V**A**SVE**T**Q~GILQGNEPMGGEPVPGISEE), encoded by StcA (AOT14_30750), revealed a perfect match to the consensus leader peptide motif (15), shown in boldface. The underlined residues in the core peptide (after the “~” symbol) are predicted to form a small, 7-residue macrolactam ring, as in xanthomonins (16).

### Nucleotide sequence accession number.

The complete genome sequence of *Stenotrophomonas acidaminiphila* strain ZAC14D2_NAIMI4_2 is available from GenBank under accession number CP012900.
